# Dietary Curcumin Protects Common Carp (*Cyprinus carpio*) Against Potassium Permanganate-Induced Toxicity: Hematological, Immunological, Oxidative, and Molecular Docking Evidence

**DOI:** 10.3390/vetsci13101085

**Published:** 2026-10-13

**Authors:** Mücahit Eroğlu, Serpil Mişe Yonar, Duygu Tanrıkulu, Harun Uslu, Muhammet Enis Yonar

**Affiliations:** 1 Department of Basic Aquatic Sciences, Faculty of Fisheries, Firat University, Elazig 23200, Türkiye; meroglu44@firat.edu.tr; 2 Department of Aquaculture, Faculty of Fisheries, Firat University, Elazig 23200, Türkiye; s_mise@firat.edu.tr; 3 Department of Biology, Faculty of Arts and Sciences, Kafkas University, Kars 36100, Türkiye; duygutanrikulu@kafkas.edu.tr; 4 Department of Pharmaceutical Chemistry, Faculty of Pharmacy, Firat University, Elazig 23200, Türkiye; huslu@firat.edu.tr

**Keywords:** curcumin, fish, hematology, immunity, oxidative stress, potassium permanganate, molecular docking

## Abstract

Potassium permanganate treatment can adversely affect fish physiology despite its usefulness in aquaculture. This study evaluated the protective effects of dietary curcumin against potassium permanganate-induced disturbances in common carp (Cyprinus carpio). Fish were fed a basal diet or diets supplemented with 1%, 2%, or 4% curcumin for eight weeks, with or without subsequent exposure to potassium permanganate at 2 mg/L for 30 min daily for seven days. Hematological and immune parameters and oxidative status in the liver, kidney, spleen, and gill were assessed. Potassium permanganate exposure altered blood and immune parameters, increased lipid peroxidation, and weakened antioxidant defenses. Curcumin supplementation alleviated these effects, with protection evident at 1%, the lowest dietary inclusion level tested, and further improvements in selected parameters at 4%. Inclusion levels below 1% were not evaluated, so the minimum effective level remains unknown. Molecular docking predicted interactions between curcumin and the antioxidant enzymes SOD, CAT, GPx, and GST at structurally plausible binding sites. These computational findings were interpreted alongside the biological results as supportive, hypothesis-generating evidence rather than confirmation of functional effects on these enzymes in vivo. Overall, dietary curcumin showed potential to reduce the physiological disturbances associated with repeated potassium permanganate exposure. Further dose–response studies should evaluate inclusion levels below 1% to identify effective and economically viable supplementation rates and determine whether curcumin affects the antiparasitic or antibacterial efficacy of potassium permanganate.

## Simple Summary

Fish farmers use potassium permanganate to control parasites and harmful microorganisms, but repeated treatments can also affect fish health. In this study, we asked whether curcumin, a natural compound found in turmeric, could help reduce these unwanted effects in common carp. Fish were fed diets containing different amounts of curcumin for eight weeks before exposure to potassium permanganate. Repeated exposure affected their blood and immune responses and weakened their natural defenses against tissue damage. Fish given curcumin showed improvements in many of these measurements. Benefits were observed at 1% of the diet, the lowest amount tested, while 4% offered additional improvements in some measurements. Because smaller amounts were not tested, we could not determine the lowest amount needed for protection. Curcumin may therefore help reduce the physical toll of repeated potassium permanganate exposure. However, we did not assess whether it changes the treatment’s ability to control parasites or bacteria. Further work is needed to establish this and determine whether lower, more affordable amounts offer similar benefits before curcumin can be recommended for this purpose on fish farms.

## 1. Introduction

Aquaculture has become one of the fastest-growing sectors of animal food production and is indispensable for global food security. Nevertheless, the ongoing intensification of farming systems has substantially increased the prevalence and transmission of infectious and parasitic diseases, posing a major threat to fish health, production efficiency, and economic sustainability [1,2,3]. To minimize these losses, aquaculture operations increasingly rely on veterinary pharmaceuticals, antiparasitic agents, antibiotics, disinfectants, and other chemotherapeutic compounds as integral components of health management programs [4,5,6]. Although these interventions have contributed significantly to disease control, their intensive and sometimes indiscriminate use has raised growing concerns about environmental impacts, drug resistance, fish welfare, and treatment-associated physiological disturbances [7,8]. Consequently, current research has increasingly focused on developing more sustainable disease management strategies that preserve therapeutic efficacy while minimizing adverse biological and ecological consequences by integrating safer treatment protocols and natural bioactive compounds [9,10,11].

Among the chemotherapeutic agents routinely employed in aquaculture, potassium permanganate (KMnO_4_) remains one of the most extensively used oxidizing compounds because of its broad-spectrum antimicrobial and antiparasitic activity, low cost, and ease of application [12,13] It is widely used as a bath treatment to prevent and control external parasitic infestations, bacterial infections, fungal diseases, and waterborne pathogens in both freshwater and marine aquaculture systems [14,15,16]. In addition to its therapeutic role, potassium permanganate has also been used as a disinfectant and prophylactic agent to improve fish health and reduce disease outbreaks under intensive farming conditions [17,18] Owing to these advantages, it continues to be regarded as an indispensable component of routine health management in commercial aquaculture. However, the therapeutic efficacy of potassium permanganate is highly dependent on accurate dosing and water quality characteristics, and its safety margin is relatively narrow [19,20]. Consequently, repeated or inappropriate applications may adversely affect fish physiology and compromise fish welfare, even when potassium permanganate is applied as part of routine health management practices.

Despite its widespread therapeutic use, accumulating evidence indicates that potassium permanganate may adversely affect fish physiology when exposure conditions exceed the optimal therapeutic window. Experimental studies have shown that inappropriate concentrations or prolonged exposure to potassium permanganate can disrupt hematological homeostasis, impair innate immune function, alter blood biochemical indices, and induce oxidative stress through excessive production of reactive oxygen species [21,22,23]. Furthermore, repeated exposure has been linked to tissue damage, intestinal pathological alterations, growth depression, and disturbances in antioxidant defense mechanisms, suggesting that the therapeutic efficacy of potassium permanganate may be accompanied by undesirable physiological consequences [22,24]. In addition, potassium permanganate treatment has been reported to alter the composition of the external fish microbiome and, under certain circumstances, even increase host susceptibility to opportunistic bacterial infections, emphasizing that its indiscriminate use may compromise overall fish health rather than improve it [25]. Collectively, these findings indicate that reducing the physiological side effects associated with routine potassium permanganate treatment is an important aspect of fish health management.

Growing concerns about the safety of chemotherapeutic agents have increased interest in natural feed additives that improve fish health while minimizing treatment-associated side effects. Among the natural bioactive compounds investigated as functional feed additives, curcumin, the principal polyphenolic constituent of *Curcuma longa*, has emerged as one of the most promising candidates for improving fish health and resilience under diverse stress conditions [26,27]. Owing to its potent antioxidant, anti-inflammatory, antimicrobial, and immunomodulatory properties, dietary curcumin has been extensively evaluated in numerous fish species and has consistently demonstrated beneficial effects on growth performance, hematological characteristics, antioxidant defense, innate immunity, intestinal health, tissue integrity, and resistance to infectious diseases [28,29,30,31,32,33]. Moreover, curcumin has been shown to alleviate physiological disturbances induced by environmental contaminants and heavy metals, improve redox homeostasis, regulate immune-related signaling pathways, and enhance disease resistance by activating endogenous antioxidant mechanisms [33,34,35]. Recent investigations have further highlighted its therapeutic potential against aquatic pathogens and parasites, supporting its application as a sustainable alternative to conventional chemotherapeutic agents in modern aquaculture [36,37,38]. Collectively, these findings suggest that dietary curcumin may help reduce the adverse physiological effects associated with chemical exposure in aquaculture. Previous studies have reported antioxidant and immunomodulatory effects of dietary curcumin in fish exposed to a range of environmental, infectious, and toxicological stressors [28,29,30,31,32,33,34,35]. These findings provide a rationale for examining whether similar benefits occur during repeated exposure to potassium permanganate. Accordingly, this study evaluated whether dietary curcumin could attenuate hematological, immunological, and oxidative disturbances in common carp (*Cyprinus carpio*) subjected to repeated potassium permanganate bath treatments. We hypothesized that dietary curcumin supplementation would strengthen antioxidant defenses, preserve immune competence, and maintain hematological homeostasis, thereby improving the physiological resilience of fish exposed to potassium permanganate. The findings of this study are expected to provide novel insights into the use of functional nutritional strategies to improve the safety of therapeutic chemical applications in aquaculture and contribute to the development of more sustainable fish health management practices.

## 2. Materials and Methods

### 2.1. Experimental Fish and Husbandry Conditions

Common carp (*Cyprinus carpio*) weighing 33.0 ± 5.0 g were obtained from a commercial fish farm. After transport to the Fisheries Research Laboratory of Fırat University in Elazığ, Türkiye, the fish underwent a two-week acclimation period. Fiberglass tanks containing dechlorinated freshwater were used for both acclimation and the feeding trial. Husbandry conditions were maintained throughout these periods: continuous aeration, daily replacement of approximately 30% of the water, and daily monitoring of temperature, dissolved oxygen, and pH. Water temperature was 24 ± 1 °C, dissolved oxygen remained above 6.5 mg/L, and pH was 7.4 ± 0.3.

All fish received the commercial basal diet during acclimation. Feed was offered by hand to apparent satiation at 09:00 and 17:00 h. This feeding schedule continued during the experiment, with diet allocation as described below. Feeding ceased 24 h before sample collection in all groups.

All experimental procedures involving animals were conducted in accordance with the guidelines of the Fırat University Animal Experiments Local Ethics Committee (Approval No. 2026/14-06, Date: 14 July 2026).

### 2.2. Experimental Diets

The basal feed was an extruded trout grower diet marketed as Ecobio (Cagatay Oil and Feed Products Inc., Izmir, Türkiye). Before supplementation, the pellets were milled to a fine powder. Curcumin (Sigma-Aldrich, St. Louis, MO, USA) was dissolved in a small volume of ethanol and then diluted with distilled water. This preparation was incorporated into the powdered feed at inclusion levels of 1%, 2%, or 4% (*w*/*w*). Additional distilled water was used to form a uniform dough for processing in a laboratory pelletizer. The resulting pellets were air-dried for 48 h at room temperature to allow ethanol evaporation, then packed in sealed polyethylene bags and refrigerated at 4 °C. The unsupplemented diet underwent the same preparation steps and received corresponding quantities of ethanol and distilled water.

The inclusion levels followed those examined in our earlier eight-week feeding study in rainbow trout (*Oncorhynchus mykiss*), in which dietary curcumin improved hematological, immune, and antioxidant parameters [35]. Using this range in common carp allowed us to examine whether increasing curcumin supplementation provided additional protection against potassium permanganate exposure. These concentrations were chosen for experimental comparison and should not be interpreted as established optimal levels or commercial feeding recommendations for common carp.

### 2.3. Experimental Design

After acclimation, 240 fish were randomly distributed among eight treatment groups. Each group comprised three replicate tanks with 10 fish each, for a total of 30 fish per treatment. During the eight-week feeding phase, the Control and PP groups received the basal diet, whereas the other groups received diets containing 1%, 2%, or 4% curcumin. Each supplemented diet was evaluated both without subsequent potassium permanganate exposure (Cur1, Cur2, and Cur4) and with exposure (Cur1+PP, Cur2+PP, and Cur4+PP). Environmental conditions and routine husbandry were consistent across tanks. Blood and tissue samples were obtained from every fish at the end of the experiment and analyzed individually.

The bath phase began after the eight-week feeding period. Fish in PP, Cur1+PP, Cur2+PP, and Cur4+PP were placed individually in separate aerated containers for a daily 30 min exposure to 2 mg/L potassium permanganate. Each fish was returned to its original tank immediately afterward. Baths were administered on seven consecutive days. Fish in Control, Cur1, Cur2, and Cur4 underwent equivalent daily transfers and spent 30 min in identical containers containing clean freshwater to account for handling and transfer effects. Throughout this seven-day phase, every group received the basal commercial feed without added curcumin; supplementation was confined to the preceding eight weeks. Feed was withheld during the final 24 h before sampling.

The exposure concentration of 2 mg/L was informed by previous reports of potassium permanganate use for treatment in freshwater aquaculture [14,39]. A 30 min bath duration was selected to represent a short exposure while limiting prolonged contact with the oxidant, with reference to earlier fish bath-treatment studies [21,23].

### 2.4. Blood and Tissue Sampling

Sampling occurred 24 h after the last potassium permanganate bath. The non-exposed groups were sampled at the same interval after their final freshwater handling procedure. No feed was offered to any group during this interval. Before sampling, fish were anesthetized with 25 mg/L benzocaine. Blood obtained by severing the caudal peduncle was divided between EDTA-coated tubes for hematology and plasma preparation and plain tubes for serum collection.

Hematological measurements were performed immediately on the EDTA-treated blood. The remaining portion was centrifuged for 10 min at 3500 rpm, and the separated plasma was placed in sterile microcentrifuge tubes and frozen at −80 °C for subsequent immunological assays. Blood in the plain tubes was first allowed to clot at room temperature. Serum was then separated using the same centrifugation conditions, transferred to sterile microcentrifuge tubes, and stored at −80 °C for biochemical and innate immune measurements.

Following blood collection, the liver, kidney, spleen, and gills were aseptically removed while the fish remained under anesthesia. Residual blood was washed from the tissues with ice-cold physiological saline (0.9% NaCl). The samples were gently dried with filter paper, wrapped separately in aluminum foil, and kept at −80 °C until preparation for oxidative stress and antioxidant enzyme assays.

### 2.5. Hematological Analyses

Red blood cell (RBC) and white blood cell (WBC) counts were obtained using Natt and Herrick’s diluent [40]. Hemoglobin (Hb) was measured with Drabkin’s reagent using the cyanmethemoglobin procedure, while hematocrit (Ht) was assessed by microhematocrit centrifugation [41]. The erythrocyte indices—mean corpuscular volume (MCV), mean corpuscular hemoglobin (MCH), and mean corpuscular hemoglobin concentration (MCHC)—were calculated following the procedures reported by Sağlam and Yonar [42] and Sağlam et al. [43].

### 2.6. Immunological Analyses

The immune assessment included white blood cell (WBC) counts, respiratory burst activity measured by nitroblue tetrazolium (NBT) reduction, phagocytic activity (PA), phagocytic index (PI), total plasma protein (TP), and immunoglobulin M (IgM). Serum measurements included bactericidal activity (BA), lysozyme activity (LYZ), and myeloperoxidase activity (MPO). WBC counting is described in the preceding section.

Respiratory burst activity was assessed through nitroblue tetrazolium (NBT) reduction, following Siwicki et al. [44] and Ispir and Yonar [45]. Fresh blood and 0.2% NBT solution were combined in equal volumes (0.1 mL) and left at room temperature for 30 min. A 0.05-mL aliquot was then added to 1.0 mL of N,N-dimethylformamide. After centrifugation, the supernatant was collected for absorbance measurement at 620 nm.

PA and PI were assessed following Siwicki and Anderson [46]. A 0.1-mL sample of fresh EDTA-anticoagulated blood was combined with 0.1 mL of bacterial suspension containing 1 × 10^7^ cells mL^−1^. After 30 min of incubation, smears were prepared on glass slides, air-dried, fixed with ethanol, and stained with Giemsa. At least 100 phagocytic cells per sample were examined by light microscopy. PA represented the percentage of these cells containing engulfed bacteria, while PI expressed the mean number of bacteria engulfed per phagocytic cell.

Total plasma protein concentration was determined using the Biuret method according to Siwicki et al. [44] and Yonar et al. [47]. Total plasma immunoglobulin (IgM) levels were estimated after selective precipitation of immunoglobulins with 12% polyethylene glycol (PEG), and IgM concentrations were calculated from the difference between total plasma protein concentrations measured before and after precipitation [44,47].

BA was assessed using a modified version of the procedure reported by Kajita et al. [48]. Each assay combined 100 μL of serum with 100 μL of bacterial suspension. Following a 1 h incubation at 25 °C, the samples were diluted in sterile phosphate-buffered saline (PBS) and plated on tryptic soy agar (TSA). Colonies were counted after 24 h of incubation to assess bacterial survival.

Serum LYZ was assayed with *Micrococcus lysodeikticus* as the substrate, following Ellis [49]. The assay tracked the decline in absorbance at 450 nm. Activity was expressed using the definition of one unit as a 0.001 decrease in absorbance per minute per milliliter of serum.

Serum MPO was measured using the Quade and Roth procedure [50], as modified by Sahoo et al. [51]. Serum was combined with 3,3′,5,5′-tetramethylbenzidine (TMB) and hydrogen peroxide (H_2_O_2_). Following termination of the reaction with 4 M sulfuric acid, absorbance was read at 450 nm on a microplate reader.

### 2.7. Oxidative Stress and Antioxidant Enzyme Analyses

For biochemical analysis, liver, kidney, spleen, and gill samples were homogenized in chilled 1.15% KCl at a tissue-to-solution ratio of 1:10 (*w*/*v*), using a glass homogenizer. The homogenates underwent centrifugation for 10 min at 3200 rpm and 4 °C. The supernatant fractions were then used for the assays described below.

Tissue malondialdehyde (MDA), used as an indicator of lipid peroxidation, was quantified through the thiobarbituric acid (TBA) reaction following Placer et al. [52]. Reduced glutathione (GSH) was measured by the Ellman procedure [53]. Spectrophotometric assays were used for the antioxidant enzymes: the protocols of Sun et al. [54], Aebi [55], Beutler [56], and Habig et al. [57] were applied to superoxide dismutase (SOD), catalase (CAT), glutathione peroxidase (GPx), and glutathione S-transferase (GST), respectively. Enzyme activities were normalized to tissue homogenate protein concentrations measured using the Lowry assay [58].

### 2.8. Molecular Docking Studies

Curcumin was modeled from its PubChem SMILES representation (https://pubchem.ncbi.nlm.nih.gov/, accessed on 5 September 2026), followed by energy minimization in ChemOffice 2015. Protein coordinates were downloaded from the Protein Data Bank (https://www.rcsb.org/, accessed on 5 September 2026) and prepared and optimized in Maestro Release 2026, version 14.7 [59]. The selected structures were SOD (3LSU), CAT (1DGB), GPx (2F8A), and GST (1F2E). Docking grids were positioned around previously identified or predicted binding regions [60], using the reported box dimensions of 60 × 60 × 60 Å and a grid spacing of 0.375 Å. AutoDock 4.2.6 calculations employed the Lamarckian Genetic Algorithm, with 50 runs per protein under standard settings. Outputs from both AutoDock and Vina 1.2.5 [61,62] are reported in the Results. As previously noted, validation through the redocking of co-crystal ligands onto their respective target sites yielded RMSD values between 1.33 and 1.81 Å for SOD, CAT, and GPx [63]. For GST, validation used its co-crystal ligand, glutathione, which yielded an RMSD of 1.22 in this study.

### 2.9. Statistical Analysis

The experiment followed a 2 × 4 factorial arrangement combining potassium permanganate exposure at 0 or 2 mg/L with dietary curcumin at 0%, 1%, 2%, or 4%. Data are summarized as means with their standard errors (SE). Distributional normality and equality of variances were checked using the Shapiro–Wilk and Levene tests, respectively.

A two-way ANOVA within the general linear model (GLM) framework evaluated the effects of potassium permanganate, curcumin inclusion level, and their interaction. Significant outcomes were followed by pairwise comparisons of treatment means using Tukey’s test. Individual blood and tissue samples collected from each fish were analyzed separately; therefore, each fish was considered the biological experimental unit for the hematological, immunological, and oxidative stress analyses. The replicate tanks were maintained under identical environmental and husbandry conditions throughout the study to minimize tank-to-tank variation.

Statistical calculations were conducted in IBM SPSS Statistics 21.0 for Windows (IBM Corp., Armonk, NY, USA), with statistical significance set at *p* < 0.05.

## 3. Results

### 3.1. Hematological Parameters

Table 1 summarizes the hematological findings. Potassium permanganate exposure reduced RBC, Hb, Ht, and MCV relative to the control group (*p* < 0.05). In fish receiving curcumin without subsequent exposure, RBC and Ht were significantly higher than control values, whereas Hb and MCV did not differ significantly from the control. All four parameters were significantly higher in Cur1, Cur2, and Cur4 than in PP. No significant differences in these parameters were detected among the three curcumin-only groups.

Fish in the Cur1+PP, Cur2+PP, and Cur4+PP groups had significantly higher RBC, Hb, and Ht values than those in the PP group (*p* < 0.05). These parameters did not differ significantly among the three curcumin-pretreated groups (*p* > 0.05), although Ht was numerically highest in Cur4+PP. MCV was significantly higher in Cur4+PP than in PP, Cur1+PP, and Cur2+PP (*p* < 0.05), whereas Cur1+PP and Cur2+PP did not differ significantly from PP. Thus, the advantage of the 4% level among the combined treatment groups was specific to MCV rather than consistent across the hematological parameters.

MCH did not differ significantly among groups (*p* > 0.05). For MCHC, the PP group had significantly higher values than the control, whereas Cur1, Cur2, and Cur4 had significantly lower values than both control and PP (*p* < 0.05). MCHC in the three curcumin-pretreated groups did not differ significantly from either control or PP.

### 3.2. Immune Response

The effects of dietary curcumin supplementation and potassium permanganate exposure on immune response parameters are summarized in Table 2. Fish in the PP group exhibited significantly higher WBC, NBT, and MPO values than those in the control group (*p* < 0.05). Conversely, PA, PI, TP, IgM, BA, and LYZ were significantly reduced following potassium permanganate exposure, indicating a marked alteration in the immune profile.

Dietary curcumin supplementation alone (Cur1, Cur2, and Cur4) significantly improved most immune-related parameters compared with the control and PP groups. Fish receiving curcumin exhibited higher PA, PI, TP, IgM, BA, and LYZ values, whereas WBC, NBT, and MPO remained significantly lower than those observed in the PP group (*p* < 0.05). No significant differences were detected among the three dietary curcumin levels for any of the evaluated immune variables.

In the Cur1+PP, Cur2+PP, and Cur4+PP groups, WBC, NBT, and MPO values were significantly lower, whereas PA, PI, TP, IgM, BA, and LYZ values were significantly higher than those in the PP group (*p* < 0.05). Recovery differed across parameters. IgM and LYZ did not differ significantly from control values in any of the three curcumin-pretreated groups (*p* > 0.05). However, WBC, NBT, and MPO remained significantly above control values, while PA, PI, TP, and BA remained significantly below them (*p* < 0.05), indicating partial recovery of these responses. No significant differences were detected among Cur1+PP, Cur2+PP, and Cur4+PP for any measured immune parameter (*p* > 0.05).

### 3.3. Oxidative Status in Different Tissues

The effects of dietary curcumin supplementation and potassium permanganate exposure on oxidative status in different tissues are presented in Table 3, Table 4, Table 5 and Table 6. Compared with the control group, fish in the PP group exhibited significantly higher MDA levels together with significantly lower SOD, CAT, GPx, GST, and GSH values in the liver, kidney, spleen, and gill (*p* < 0.05), indicating a consistent disturbance of tissue redox homeostasis following potassium permanganate exposure.

Differences among Cur1, Cur2, and Cur4 depended on the tissue and parameter examined (Table 3, Table 4, Table 5 and Table 6). For example, hepatic GPx and GSH were significantly higher in Cur4 than in Cur1 and Cur2, whereas hepatic SOD and CAT did not differ significantly between Cur2 and Cur4. Kidney SOD and CAT were significantly higher in Cur4 than in the other two supplemented groups, but kidney GSH did not differ significantly among the three levels. Likewise, no significant differences among Cur1, Cur2, and Cur4 were detected for splenic CAT or gill GST and GSH. Therefore, numerically higher antioxidant values at 4% did not consistently represent statistically significant improvements over the lower inclusion levels.

Compared with PP alone, all three curcumin pretreatments significantly reduced MDA and increased SOD, CAT, GPx, GST, and GSH in the examined tissues (*p* < 0.05). However, differences among pretreatment levels varied by parameter and tissue. Cur4+PP had significantly lower hepatic and renal MDA than Cur1+PP and Cur2+PP, whereas splenic MDA did not differ significantly between Cur2+PP and Cur4+PP. Gill MDA decreased significantly at each successive inclusion level. Hepatic CAT and GPx and gill GSH were significantly higher in Cur2+PP and Cur4+PP than in Cur1+PP, with no significant differences between the 2% and 4% levels. GST did not differ significantly among the three pretreatment groups in any tissue. Similarly, splenic CAT, GPx, and GSH and gill SOD showed no significant differences among these groups. These findings indicate that higher inclusion levels benefit selected endpoints, rather than producing a uniform improvement with increasing supplementation.

### 3.4. Molecular Docking Studies

**Figure 1:**
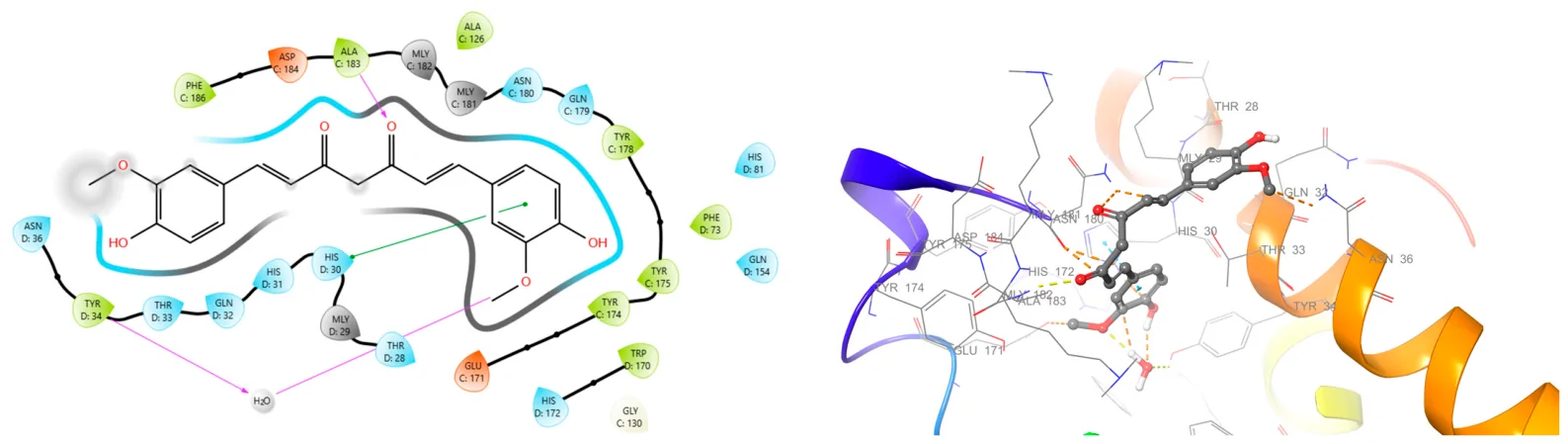
Two-dimensional and three-dimensional interaction diagrams with SOD/3LSU for curcumin.

**Figure 2:**
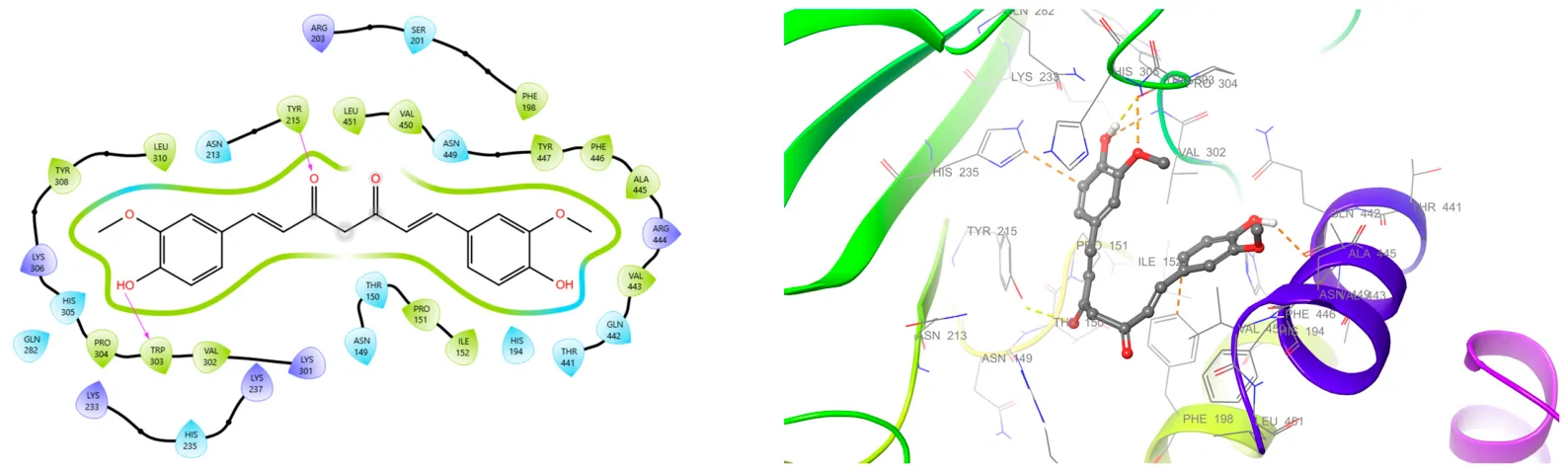
Two-dimensional and three-dimensional interaction diagrams with CAT/1DGB for curcumin.

**Figure 3:**
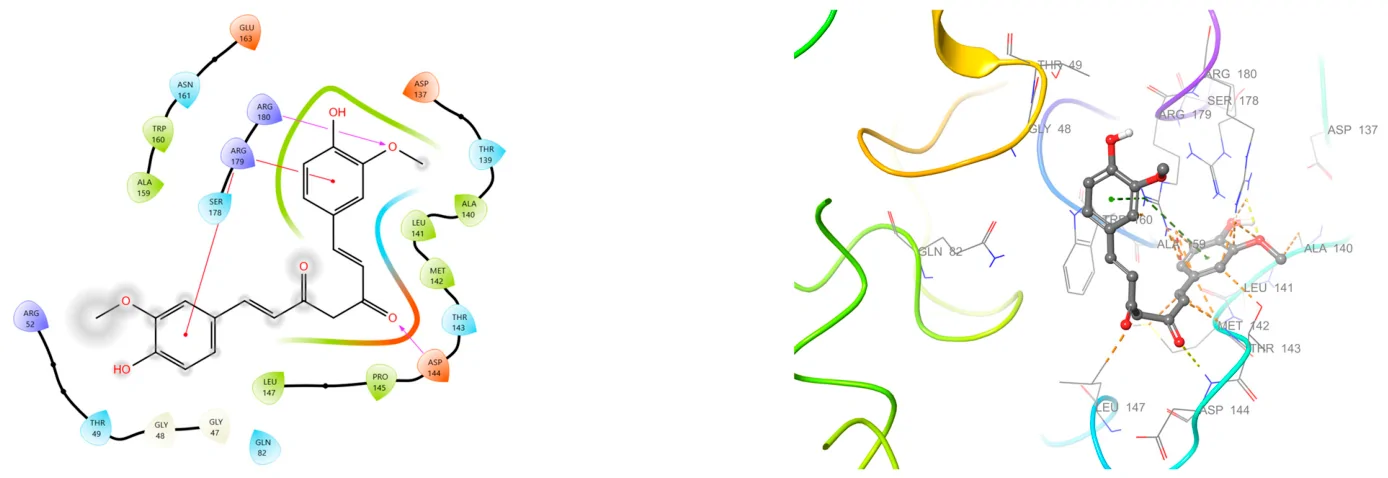
Two-dimensional and three-dimensional interaction diagrams with GPx/2F8A for curcumin.

**Figure 4:**
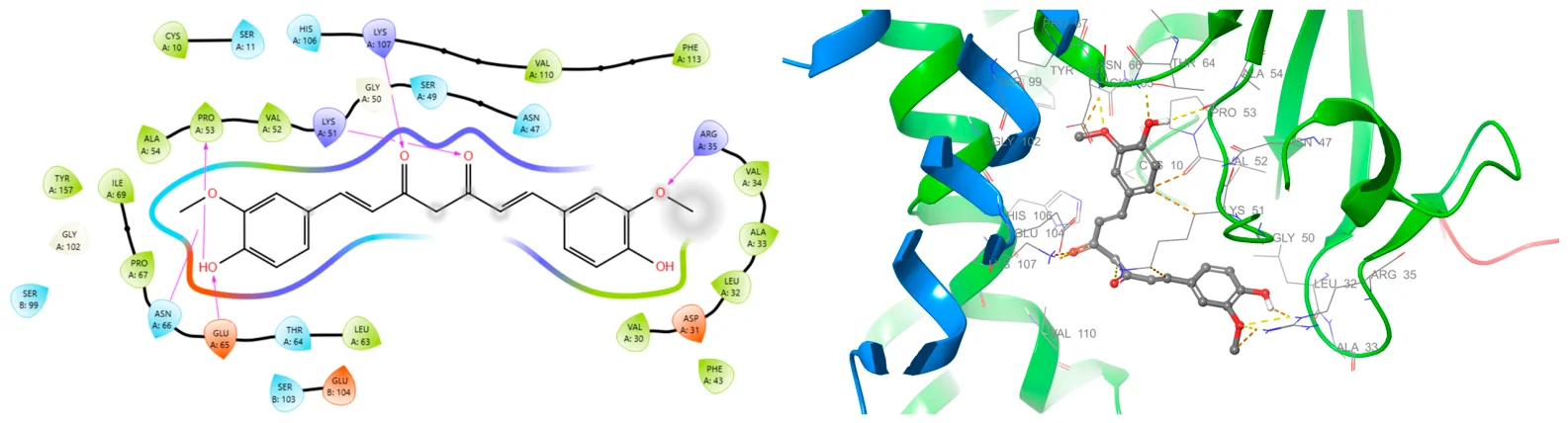
Two-dimensional and three-dimensional interaction diagrams with GST/1F2E for curcumin.

Curcumin forms hydrogen bonds with ALA183, HIS30, and TYR34 residues of SOD, and with TYR215 and TRP303 residues of CAT. For GPx, curcumin interacts with ASP144 and ARG180 through hydrogen bonds and with ARG179 through a pi-cation interaction. In addition, curcumin forms hydrogen bonds with ARG35, LYS51, PRO53, GLU65, ASN66, and LYS107 residues of GST. The estimated inhibition constants (Ki) indicated that curcumin may exhibit micromolar-level activity against SOD (4.67 µM), CAT (1.57 µM), and GST (1.13 µM), whereas its predicted activity against GPx was comparatively weaker (151.05 µM). Furthermore, the Vina docking scores were −7.2, −8.8, −5.7, and −7.9 kcal/mol for SOD, CAT, GPx, and GST, respectively (Table 7 and Figure 1, Figure 2, Figure 3 and Figure 4).

## 4. Discussion

The widespread application of potassium permanganate as a chemotherapeutic agent in aquaculture necessitates a better understanding of its physiological consequences and the development of nutritional strategies capable of minimizing treatment-associated adverse effects. In the present study, repeated potassium permanganate exposure induced marked alterations in hematological characteristics, innate immune competence, and tissue redox balance in common carp, whereas dietary curcumin supplementation substantially mitigated these disturbances under both normal and chemotherapeutic conditions. Although the degree of recovery varied among the evaluated parameters, curcumin consistently improved physiological homeostasis by preserving blood characteristics, maintaining innate immune function, and strengthening antioxidant defenses in multiple tissues. Collectively, these findings provide experimental evidence that dietary curcumin represents an effective supportive nutritional strategy for enhancing the physiological resilience of fish subjected to repeated potassium permanganate treatment, thereby contributing to safer and more sustainable health management practices in aquaculture.

To better appreciate the significance of these findings, they should be interpreted in the context of previous studies evaluating dietary curcumin under different experimental conditions. Previous studies have consistently demonstrated the beneficial effects of dietary curcumin in fish exposed to a wide range of environmental and experimental stressors, including pesticides, heavy metals, ammonia, pathogenic infections, and other toxic conditions. These investigations primarily focused on improving antioxidant capacity, immune function, growth performance, and disease resistance or on alleviating physiological disturbances induced by environmental contaminants and chemical toxicants [28,29,30,31,32,33,34,35,36,37,38]. In contrast, the present study addresses a fundamentally different research question by evaluating dietary curcumin as a supportive nutritional strategy during repeated potassium permanganate bath treatment, a routine chemotherapeutic practice widely applied in aquaculture. This study specifically evaluated whether dietary curcumin could alleviate hematological, immunological, and oxidative disturbances in common carp following repeated potassium permanganate bath exposure. Therefore, the novelty of the present study lies not in demonstrating the well-established antioxidant and immunomodulatory properties of dietary curcumin, but in establishing its potential as a supportive nutritional strategy to enhance the physiological safety of repeated potassium permanganate chemotherapeutic treatments in aquaculture. Based on this conceptual framework, the present findings are discussed below with particular emphasis on the hematological, immunological, and oxidative responses associated with repeated potassium permanganate treatment.

Potassium permanganate exposure significantly altered the hematological profile of common carp, as evidenced by reductions in RBC, Hb, Ht, and MCV together with an increase in MCHC, whereas MCH remained unaffected. These alterations suggest that repeated potassium permanganate exposure adversely influences erythrocyte homeostasis and may impair the oxygen transport capacity of the blood. As a potent oxidizing agent, potassium permanganate is capable of generating reactive oxygen species that induce lipid peroxidation of erythrocyte membranes, thereby increasing membrane fragility and reducing erythrocyte survival. Accordingly, the reductions observed in RBC, Hb, and Ht are likely associated with oxidative damage to circulating erythrocytes rather than solely with impaired erythropoiesis, whereas the unchanged MCH indicates that potassium permanganate primarily affected erythrocyte integrity and cell number without markedly altering the hemoglobin content of individual erythrocytes. Similar hematological disturbances have been reported in Nile tilapia, African catfish, and Labeo rohita following potassium permanganate exposure, where reductions in erythrocyte-related variables were accompanied by immunological and biochemical disturbances associated with oxidative stress [22,23,64]. Likewise, increased physiological stress responses following potassium permanganate exposure have been documented in channel catfish and goldfish, indicating that even therapeutic concentrations may induce measurable systemic stress and hematological disturbances [21,65]. Since hematological variables are highly sensitive indicators of fish health and physiological status [66], the present findings demonstrate that repeated potassium permanganate treatment, despite its established therapeutic value, may compromise hematological homeostasis and therefore highlights the importance of improving the physiological safety of routine chemotherapeutic applications in aquaculture.

Dietary curcumin supplementation alone improved the hematological status of common carp, as demonstrated by higher RBC, Hb, Ht, and MCV values in the Cur1, Cur2, and Cur4 groups compared with the Control group. These findings indicate that curcumin supports erythrocyte stability and blood homeostasis under non-stressed conditions, most likely through its antioxidant and membrane-protective properties. Similar hematological improvements have been reported in rainbow trout [35], common carp [67,68,69], gilthead seabream, and Nile tilapia [30,70,71], demonstrating that dietary curcumin contributes to the maintenance of normal blood physiology under both normal and stressful conditions. In the present study, most hematological variables showed comparable responses among Cur1, Cur2, and Cur4, with beneficial responses already evident at 1%, the lowest dietary inclusion level tested.

The partial restoration of RBC, Hb, Ht, and MCV observed in the Cur1+PP, Cur2+PP, and Cur4+PP groups demonstrates that dietary curcumin effectively alleviated the hematological disturbances induced by repeated potassium permanganate exposure. Although these variables were not completely restored to the levels observed in the curcumin-only groups, their significant improvement relative to the PP group indicates that curcumin preserved hematological homeostasis during repeated chemotherapeutic treatment. This protective effect is likely associated with the ability of curcumin to reduce oxidative damage to erythrocyte membranes, maintain membrane stability, and protect hematopoietic tissues, thereby improving erythrocyte survival and preserving the oxygen-carrying capacity of blood. Previous studies have similarly demonstrated that dietary curcumin ameliorates hematological disturbances and improves physiological status in common carp [67,68,69], rainbow trout [35], Nile tilapia, European seabass, and other cultured fish species [70,71,72]. However, unlike previous investigations that primarily evaluated dietary curcumin under pesticide, toxicant, pathogen, or other environmental stress conditions, the present study specifically demonstrates its protective role during repeated potassium permanganate bath treatment, one of the most widely used chemotherapeutic procedures in aquaculture. Therefore, the present findings support further investigation of dietary curcumin as a nutritional strategy to mitigate potassium permanganate-associated hematological disturbances, while suitable supplementation rates for practical use remain to be established. Potassium permanganate exposure markedly disrupted the innate immune system of common carp, as evidenced by significant increases in WBC, NBT, and MPO, accompanied by marked reductions in PA, PI, TP, IgM, BA, and LYZ. These findings suggest that repeated potassium permanganate treatment elicited an imbalance between immune cell activation and functional immune competence. The elevated WBC count may reflect stress-induced leukocyte mobilization, whereas increased NBT and MPO activities indicate enhanced respiratory burst and neutrophil activation in response to oxidative challenge. However, the concurrent reductions in PA and PI demonstrate that despite activation of oxidative defense mechanisms, the phagocytic capacity of immune cells was compromised. Likewise, decreases in BA, LYZ, and IgM indicate impairment of both humoral antimicrobial defense and innate immune effectiveness, suggesting that prolonged oxidant exposure shifted the immune response from a protective state toward physiological dysfunction. These findings agree with previous studies demonstrating that potassium permanganate treatment adversely affects immune regulation in aquatic organisms. Alterations in leukocyte profiles and immune-related parameters have been reported in *Labeo rohita* [23], while reduced disease resistance and increased susceptibility to bacterial infections have been described in giant freshwater prawn and fish following potassium permanganate exposure [25,73]. Similar physiological stress responses have also been documented in channel catfish and goldfish [21,65]. Because oxidative stress is closely associated with impairment of innate immune functions during repeated environmental or chemotherapeutic challenges [74,75], the present findings indicate that repeated potassium permanganate treatment may compromise immune homeostasis and increase susceptibility to opportunistic infections despite its recognized therapeutic value. These observations further emphasize the need to improve the physiological safety of routine chemotherapeutic applications in aquaculture.

Dietary curcumin supplementation alone significantly improved the innate immune status of common carp, as evidenced by increased PA, PI, TP, IgM, BA, and LYZ together with lower WBC, NBT, and MPO values than those observed in the PP group. These findings indicate that curcumin enhanced immune competence while preventing excessive inflammatory and oxidative activation of immune cells, thereby maintaining immune homeostasis under normal physiological conditions. Similar immunostimulatory effects have been reported in Nile tilapia [28], rainbow trout [35], common carp, European seabass, Nile tilapia, and snakehead fish [68,70,72,76]. Dietary curcumin has also been shown to strengthen mucosal immunity, regulate immune-related signaling pathways, including NF-κB, and improve disease resistance in cultured fish [29,77,78,79]. In addition, long-term dietary supplementation has been reported to regulate inflammatory cytokine expression while preserving immune homeostasis [31,71,80]. Collectively, these studies support the role of dietary curcumin as an effective immunomodulatory feed additive that enhances innate immune competence without inducing excessive oxidative immune activation.

Curcumin pretreatment substantially mitigated the immune disturbances induced by potassium permanganate, as shown by lower WBC, NBT, and MPO values and higher PA, PI, TP, IgM, BA, and LYZ levels in the Cur1+PP, Cur2+PP, and Cur4+PP groups compared with the PP group. This response indicates that curcumin did not merely suppress immune activation but rather restored immune homeostasis by limiting excessive oxidative responses while preserving functional antimicrobial defense. The reduction in NBT and MPO suggests attenuation of excessive respiratory burst activity, whereas the recovery of PA, PI, IgM, BA, and LYZ demonstrates improved cellular and humoral immune competence during repeated potassium permanganate exposure. Previous studies have likewise shown that dietary curcumin improves innate immunity, immunoglobulin production, lysozyme activity, phagocytic function, antioxidant capacity, and disease resistance in fish exposed to infectious, environmental, or toxicological stressors [28,34,35,68]. Similar beneficial responses have also been reported in Nile tilapia, European seabass, common carp, and greater amberjack receiving dietary curcumin or curcumin nanoparticles [70,71,72,78]. However, unlike previous investigations that primarily evaluated curcumin under infectious, toxicological, or environmental stress conditions, the present study demonstrates its ability to preserve innate immune homeostasis during repeated potassium permanganate bath treatments, one of the most widely used chemotherapeutic procedures in aquaculture. Taken together, the present findings suggest that dietary curcumin enhances the immunological resilience of fish during repeated potassium permanganate treatment by preserving innate immune competence and antimicrobial defense, thereby reducing treatment-associated immune dysfunction in aquaculture.

Potassium permanganate exposure induced a consistent oxidative imbalance in all examined tissues, as reflected by elevated MDA levels and concomitant reductions in SOD, CAT, GPx, GST, and GSH. This response indicates that repeated potassium permanganate treatment enhanced lipid peroxidation while weakening both enzymatic and non-enzymatic antioxidant defenses. As a strong oxidizing compound, potassium permanganate promotes excessive ROS generation, leading to membrane lipid peroxidation and depletion of endogenous antioxidant systems. The reductions in SOD and CAT indicate impaired scavenging of superoxide radicals and hydrogen peroxide, whereas the decreases in GPx, GST, and GSH reflect disruption of the glutathione-dependent antioxidant and detoxification system. Similar oxidative disturbances have been reported following potassium permanganate exposure in Nile tilapia, where increased oxidative damage and impaired antioxidant defenses were observed [22,24,81]. In addition, potassium permanganate and related oxidizing compounds have been associated with oxidative stress, endocrine disturbances, and neurological impairment in fish [82,83]. Since oxidative stress develops when ROS production exceeds endogenous antioxidant capacity [84,85], the present findings demonstrate that repeated potassium permanganate treatment compromises tissue redox homeostasis and may reduce the physiological resilience of fish despite its recognized therapeutic value. These observations further highlight the need to improve the biological safety of routine potassium permanganate applications in aquaculture.

Dietary curcumin supplementation alone markedly improved the oxidative status of common carp, as shown by reduced MDA levels and significant increases in SOD, CAT, GPx, GST, and GSH in all examined tissues. These findings indicate that curcumin effectively strengthened both enzymatic and non-enzymatic antioxidant defense systems, thereby maintaining tissue redox homeostasis under normal physiological conditions. Similar antioxidant responses have been consistently reported in common carp following malathion and chlorpyrifos exposure [67,86] in rainbow trout [35], and in Nile tilapia, gilthead seabream, red tilapia, and common carp receiving dietary curcumin [31,32,68,87]. More recent studies have also shown that curcumin alleviates oxidative injury induced by ammonia, chromium, and other environmental stressors by enhancing endogenous antioxidant defenses [88,89,90]. Previous studies have implicated Nrf2-related signaling in the antioxidant effects of curcumin [91,92]. This pathway may explain the improvements in antioxidant status observed here; however, the present study did not assess Nrf2 activation or downstream gene expression. Collectively, these findings confirm that dietary curcumin effectively reinforces endogenous antioxidant capacity and preserves tissue redox balance.

Dietary curcumin supplementation markedly alleviated potassium permanganate-induced oxidative damage, as demonstrated by the significant reduction in MDA levels together with restoration of SOD, CAT, GPx, GST, and GSH in the Cur1+PP, Cur2+PP, and Cur4+PP groups. Although antioxidant parameters were not completely restored to the levels observed in the curcumin-only groups, their substantial recovery relative to the PP group indicates that curcumin effectively preserved tissue redox homeostasis during repeated chemotherapeutic exposure. Restoration of the glutathione-dependent antioxidant system, together with reduced lipid peroxidation, suggests that curcumin enhanced tissues’ capacity to neutralize ROS and limit oxidative injury. Previous studies similarly demonstrated that dietary curcumin restored antioxidant defenses in common carp exposed to malathion and chlorpyrifos [67,86], alleviated lead-induced oxidative stress [34], and protected fish against oxidative injury induced by ammonia, chromium, and other environmental stressors [88,89,90]. Comparable improvements have also been reported in Nile tilapia and other cultured fish species receiving dietary curcumin [31,68,87]. Although previous studies suggest that Nrf2-related regulation of antioxidant and phase II detoxification enzymes may contribute to the protective effects of curcumin [91,92], the biochemical changes observed in this study cannot establish the involvement of this pathway. However, unlike previous studies that primarily evaluated curcumin under environmental or toxicological stress conditions, the present study specifically demonstrates its effectiveness in preserving tissue redox homeostasis during repeated potassium permanganate bath treatment, one of the most frequently applied chemotherapeutic procedures in aquaculture. Overall, these findings indicate that dietary curcumin improves the redox stability of fish during repeated potassium permanganate treatment by limiting oxidative damage and reinforcing endogenous antioxidant defenses, thereby supporting safer therapeutic use of potassium permanganate in aquaculture.

The three dietary inclusion levels should also be considered for their potential practical use. The 1% curcumin diet provided significant protection against potassium permanganate-associated alterations in most measured parameters, whereas increasing supplementation to 4% produced additional improvements in selected indices rather than a consistent advantage across all endpoints. In particular, no significant differences among the three curcumin-pretreated groups were detected for the measured immune parameters or for RBC, Hb, and Ht. Although Ht was numerically higher at 4%, this difference was not statistically significant. Significant advantages at higher inclusion levels were restricted to particular endpoints, including MCV and selected tissue oxidative stress markers. The results therefore do not support a consistently superior protective effect of the highest inclusion level. Thus, 1% was the lowest dietary inclusion level tested that showed protective effects under the present experimental conditions. Since inclusion levels below 1% were not evaluated, this finding does not establish the true minimum effective level or a recommended supplementation rate for practical aquaculture. The 4% inclusion level corresponds to 40 g curcumin/kg feed and raises practical concerns regarding ingredient costs and feed formulation. The additional physiological benefits observed at this level do not, by themselves, justify its use under commercial farming conditions. As growth performance, feed utilization, and economic feasibility were not assessed, further dose–response trials should evaluate inclusion levels below 1% to determine whether comparable protection can be achieved at lower cost and to identify suitable supplementation rates for practical application.

The crystal structure of SOD with its ligand glycerol (PDB: GOL) has been determined, and the ligand interaction domain has been clearly characterized (https://www.ebi.ac.uk/pdbe/entry/pdb/3lsu?activeTab=ligands&id=GOL, accessed on 5 September 2026). As we also pointed out in our previous studies, TYR178, MLY181, ASP184, and Na209 are important residues in terms of ligand interactions [63,93]. In the present study, it was determined that our ligand, curcumin, interacts with the same region of SOD in a similar manner. Notably, curcumin was observed to form a hydrogen bond with C: ALA183 and D: TYR34, also pi-pi stacking with D: HIS30. However, curcumin was found not to interact with residues that form hydrogen bonds with glycerol (PDB: GOL). Furthermore, curcumin was computationally predicted to inhibit SOD at the micromolar level.

The crystal structure of CAT with its ligand NADPH (PDB: NDP) has been determined, and the ligand interaction domain has been clearly characterized (https://www.ebi.ac.uk/pdbe/entry/pdb/1dgb?activeTab=ligands&id=NDP, accessed on 5 September 2026). As we also pointed out in our previous studies, TRP303 is one of the important residues in terms of ligand interactions [93]. In the present study, it was determined that curcumin interacts with the same region of CAT in a similar manner. Notably, curcumin was observed to form hydrogen bonds with TYR215 and TRP303 residues. Curcumin was computationally predicted to inhibit CAT at the micromolar level.

The crystal structure of GPx with its ligand malonic acid (PDB: MLA501) has been determined, and the ligand interaction domain has been clearly characterized (https://www.ebi.ac.uk/pdbe/entry/pdb/2f8a?activeTab=ligands, accessed on 5 September 2026). As we also pointed out in our previous studies, ASP144 and ARG179 are some of the important residues in terms of ligand interactions [93]. In the present study, it was determined that curcumin interacts with the same region of GPx in a similar manner. Notably, curcumin was observed to form hydrogen bonds with ASP144 and ARG180, while ARG179 was involved in a pi-cation interaction. Curcumin was computationally predicted to inhibit GPx at the micromolar level.

The crystal structure of GST (PDB ID: 1F2E) with its ligand glutathione (PDB ID: GSH) has been determined, and the ligand interaction domain has been clearly characterized (https://www.ebi.ac.uk/pdbe/entry/pdb/1f2e?activeTab=ligands&id=GSH, accessed on 5 September 2026). For glutathione S-transferase (GST) and the glutathione complex, when important covalent and hydrogen-bond interactions are evaluated, the amino acids CYS10, VAL52, and ASN66 stand out in particular. CYS10 is the most noteworthy because of its covalent and polar interactions with the ligand. It has also been noted that hydrogen bonds and polar interactions are observed between CYS10 and the ligand. VAL52 exhibits two significant hydrogen bonds and polar interactions with the ligand. ASN66 forms two hydrogen bonds and polar interactions with the ligand. Based on these results, it has been concluded that the amino acids CYS10, VAL52, and ASN66 play a significant role in the binding of glutathione. In the present study, it was determined that curcumin interacts with the same region of GST in a similar manner. Notably, curcumin formed hydrogen bonds with ARG35, LYS51, PRO53, GLU65, ASN66, and LYS107. Curcumin was computationally predicted to inhibit GST at the micromolar level.

These computational modeling and docking results are not definitive proof, but only present a testable hypothesis. The study did not experimentally measure the direct effects of curcumin on the catalytic activity of purified SOD, CAT, GPx, or GST en-zymes, or their interactions in the living organism (in vivo). Molecular docking often uses a simplified representation of protein conformational flexibility and cannot carefully account for the dynamic cellular environment, solvent effects, competing endogenous ligands, protein–protein interactions, cellular uptake, and metabolism. Therefore, the estimated Ki values mentioned here are computational estimates, not experimentally determined inhibition constants. Therefore, these scores do not prove that the antioxidant effects observed in fish occur through direct binding to or inhibition of these enzymes; they only provide a possible structural explanation for how curcumin might interact with them.

A key limitation of this study is that we did not evaluate the antiparasitic and antibacterial efficacy of potassium permanganate. The improvements in physiological and immune parameters, including serum bactericidal activity, do not establish whether dietary curcumin affects the therapeutic efficacy of potassium permanganate. Therefore, these findings support a potential role for curcumin in mitigating exposure-associated physiological disturbances but cannot demonstrate that treatment efficacy is preserved. Studies using infected or parasitized fish and directly assessing pathogen or parasite clearance are needed to determine whether these physiological benefits can be achieved without compromising treatment outcomes.

A further limitation concerns the interpretation of the mechanisms underlying the observed protection. The hematological, immunological, and oxidative stress measurements document physiological responses but do not identify the signaling pathways responsible for them. Expression of Nrf2 and downstream antioxidant- and immune-related genes, as well as protein-level markers of pathway activation, was not assessed. Therefore, Nrf2 involvement and other signaling mechanisms discussed here should be considered potential explanations based on previous studies rather than mechanisms demonstrated by our experiments. The molecular docking analyses likewise do not establish activation of these pathways in vivo. Future studies combining gene-expression analyses with protein-level measurements, including assessment of Nrf2 nuclear translocation, would help test these mechanistic hypotheses.

## 5. Conclusions

This study shows that repeated potassium permanganate exposure induces significant hematological, immunological, and oxidative disturbances in common carp, indicating that although potassium permanganate remains an effective chemotherapeutic agent, repeated treatments may compromise physiological homeostasis. Dietary curcumin supplementation substantially alleviated these adverse effects by preserving hematological integrity, maintaining innate immune competence, and reinforcing tissue antioxidant defense systems. The coordinated reduction in lipid peroxidation together with the restoration of endogenous antioxidant mechanisms indicates that curcumin enhances the physiological resilience of fish during repeated potassium permanganate treatment. Computational docking identified predicted binding interactions between curcumin and SOD, CAT, GPx, and GST, which may provide a potential structural rationale for the observed antioxidant responses. Under the present experimental conditions, 1% was the lowest dietary inclusion level tested that showed protective effects. Because lower inclusion levels were not evaluated, 1% should not be interpreted as the true minimum effective level or a practical recommended dose. Although 4% provided additional benefits in selected indices, its economic feasibility remains unassessed. Further dose–response trials with inclusion levels below 1%, together with assessments of growth, feed utilization, and supplementation costs, are needed to identify suitable rates for practical aquaculture. Under the experimental conditions of the present study, dietary curcumin showed potential to mitigate the physiological disturbances associated with repeated potassium permanganate exposure in common carp. However, whether this supplementation affects the antiparasitic or antibacterial efficacy of potassium permanganate remains unknown. Further studies should assess both physiological responses and treatment efficacy in disease-treatment models before making practical recommendations.

## Figures and Tables

**Table 1:** Effects of dietary curcumin supplementation and potassium permanganate exposure on hematological parameters.

		Groups							
		Control	PP	Cur1	Cur2	Cur4	Cur1+PP	Cur2+PP	Cur4+PP
Parameters	RBC	1.53 ± 0.26 ^b^	1.18 ± 0.33 ^d^	1.78 ± 0.20 ^a^	1.80 ± 0.36 ^a^	1.82 ± 0.29 ^a^	1.42 ± 0.14 ^c^	1.44 ± 0.23 ^c^	1.45 ± 0.18 ^c^
	Hb	6.95 ± 0.35 ^a^	5.19 ± 0.24 ^c^	7.05 ± 0.15 ^a^	7.08 ± 0.19 ^a^	7.10 ± 0.13 ^a^	6.19 ± 0.29 ^b^	6.24 ± 0.36 ^b^	6.27 ± 0.30 ^b^
	Ht	33.18 ± 3.24 ^c^	21.01 ± 2.41 ^d^	42.66 ± 2.91 ^a^	43.05 ± 3.48 ^a^	43.87 ± 3.10 ^a^	27.14 ± 1.95 ^b^	27.36 ± 2.41 ^b^	29.99 ± 2.17 ^bc^
	MCV	215.22 ± 8.75 ^ab^	176.05 ± 6.91 ^c^	236.66 ± 7.82 ^a^	235.17 ± 9.41 ^a^	239.27 ± 8.33 ^a^	190.18 ± 9.98 ^c^	188.06 ± 7.06 ^c^	205.73 ± 6.77 ^b^
	MCH	44.62 ± 3.74 ^a^	44.71 ± 2.98 ^a^	40.88 ± 3.10 ^a^	38.53 ± 4.21 ^a^	39.01 ± 2.47 ^a^	44.59 ± 3.22 ^a^	43.53 ± 2.70 ^a^	43.85 ± 3.18 ^a^
	MCHC	20.86 ± 2.17 ^b^	24.82 ± 1.76 ^a^	16.49 ± 1.45 ^c^	16.71 ± 2.05 ^c^	16.19 ± 1.82 ^c^	22.86 ± 1.37 ^ab^	22.47 ± 2.23 ^ab^	21.01 ± 1.65 ^ab^

Control: fish fed the basal diet without potassium permanganate exposure; PP: fish fed the basal diet and exposed to potassium permanganate; Cur1: fish fed a diet supplemented with 1% curcumin; Cur2: fish fed a diet supplemented with 2% curcumin; Cur4: fish fed a diet supplemented with 4% curcumin; Cur1+PP: fish fed a diet supplemented with 1% curcumin and subsequently exposed to potassium permanganate; Cur2+PP: fish fed a diet supplemented with 2% curcumin and subsequently exposed to potassium permanganate; Cur4+PP: fish fed a diet supplemented with 4% curcumin and subsequently exposed to potassium permanganate. RBC: Red blood cell (×10^6^); Hb: Hemoglobin (g/dL); Ht: Hematocrit (%); MCV: Mean corpuscular volume (µm^3^); MCH: Mean corpuscular hemoglobin (pg); MCHC: Mean corpuscular hemoglobin concentration (%). Values are presented as mean ± SE (*n* = 30). Different lowercase letters within the same row indicate significant differences among groups (*p* < 0.05, Tukey’s multiple comparison test).

**Table 2:** Effects of dietary curcumin supplementation and potassium permanganate exposure on immune parameters.

		Groups							
		Control	PP	Cur1	Cur2	Cur4	Cur1+PP	Cur2+PP	Cur4+PP
Parameters	WBC	37.41 ± 3.59 ^d^	52.24 ± 5.08 ^a^	42.18 ± 3.94 ^bc^	43.45 ± 2.88 ^bc^	45.18 ± 2.47 ^c^	41.19 ± 2.41 ^b^	42.55 ± 2.73 ^bc^	40.19 ± 3.20 ^b^
	NBT	1.24 ± 0.20 ^d^	1.55 ± 0.17 ^a^	1.33 ± 0.12 ^c^	1.35 ± 0.15 ^c^	1.36 ± 0.13 ^c^	1.47 ± 0.12 ^b^	1.45 ± 0.14 ^b^	1.44 ± 0.13 ^b^
	PA	34.50 ± 2.75 ^b^	22.35 ± 2.20 ^d^	42.25 ± 3.85 ^a^	43.58 ± 3.70 ^a^	43.24 ± 2.45 ^a^	27.33 ± 2.51 ^c^	28.42 ± 3.02 ^c^	29.10 ± 2.97 ^c^
	PI	3.33 ± 0.27 ^b^	2.20 ± 0.14 ^d^	4.47 ± 0.15 ^a^	4.56 ± 0.21 ^a^	4.60 ± 0.16 ^a^	2.68 ± 0.12 ^c^	2.72 ± 0.13 ^c^	2.75 ± 0.10 ^c^
	TP	29.26 ± 3.61 ^b^	20.14 ± 2.41 ^d^	35.76 ± 3.02 ^a^	36.10 ± 2.52 ^a^	36.84 ± 3.22 ^a^	24.89 ± 2.37 ^c^	25.10 ± 3.06 ^c^	25.55 ± 2.13 ^c^
	IgM	14.10 ± 2.40 ^b^	8.79 ± 1.88 ^c^	16.79 ± 2.04 ^a^	17.41 ± 1.59 ^a^	17.80 ± 1.91 ^a^	12.93 ± 1.11 ^b^	13.04 ± 1.46 ^b^	13.13 ± 1.70 ^b^
	BA	40.19 ± 5.52 ^b^	21.63 ± 4.12 ^d^	54.87 ± 3.96 ^a^	55.29 ± 5.03 ^a^	57.66 ± 4.71 ^a^	33.12 ± 3.16 ^c^	34.00 ± 2.82 ^c^	34.89 ± 2.01 ^c^
	LYZ	126.13±17.00^b^	92.75 ± 14.23^c^	149.58 ± 16.84^a^	152.23 ± 13.40^a^	155.07 ± 18.06^a^	118.93 ± 12.74^b^	121.42 ± 10.29^b^	119.64 ± 10.82^b^
	MPO	0.71 ± 0.12 ^d^	1.25 ± 0.10 ^a^	0.86 ± 0.08 ^c^	0.91 ± 0.11 ^c^	0.89 ± 0.14 ^c^	1.02 ± 0.10 ^b^	1.00 ± 0.13 ^b^	0.97 ± 0.09 ^b^

Control: fish fed the basal diet without potassium permanganate exposure; PP: fish fed the basal diet and exposed to potassium permanganate; Cur1: fish fed a diet supplemented with 1% curcumin; Cur2: fish fed a diet supplemented with 2% curcumin; Cur4: fish fed a diet supplemented with 4% curcumin; Cur1+PP: fish fed a diet supplemented with 1% curcumin and subsequently exposed to potassium permanganate; Cur2+PP: fish fed a diet supplemented with 2% curcumin and subsequently exposed to potassium permanganate; Cur4+PP: fish fed a diet supplemented with 4% curcumin and subsequently exposed to potassium permanganate. WBC: White blood cell counts (×10^3^); NBT: Nitroblue tetrazolium assay (mg/mL); PA: Phagocytic activity (%); PI: Phagocytic index; TP: Total protein level (mg/mL); IgM: Total immunoglobulin M level (mg/mL); BA: Serum bactericidal activity (% cfu/control); LYZ: Lysozyme activity (U/mL); MPO: Myeloperoxidase activity (Optic density). Values are presented as mean ± SE (*n* = 30). Different lowercase letters within the same row indicate significant differences among groups (*p* < 0.05, Tukey’s multiple comparison test).

**Table 3:** Effects of dietary curcumin supplementation and potassium permanganate exposure on lipid peroxidation and antioxidant defense system in the liver.

		Groups							
		Control	PP	Cur1	Cur2	Cur4	Cur1+PP	Cur2+PP	Cur4+PP
Parameters	MDA	1.63 ± 0.16 ^d^	3.15 ± 0.33 ^a^	1.26 ± 0.21 ^e^	1.03 ± 0.19 ^f^	1.01 ± 0.12 ^f^	2.15 ± 0.26 ^b^	2.08 ± 0.14 ^b^	1.71 ± 0.17 ^c^
	SOD	2.51 ± 0.22 ^c^	1.64 ± 0.19 ^f^	2.88 ± 0.32 ^b^	2.91 ± 0.44 ^b^	3.25 ± 0.52 ^a^	1.96 ± 0.14 ^e^	2.05 ± 0.10 ^de^	2.28 ± 0.21 ^d^
	CAT	2.87 ± 0.35 ^c^	1.26 ± 0.12 ^f^	3.02 ± 0.18 ^b^	3.07 ± 0.30 ^b^	3.59 ± 0.43 ^a^	1.84 ± 0.29 ^e^	1.93 ± 0.41 ^e^	2.36 ± 0.35 ^d^
	GPx	2.29 ± 0.14 ^c^	1.02 ± 0.11 ^f^	2.58 ± 0.20 ^b^	2.93 ± 0.16 ^a^	3.00 ± 0.29 ^a^	1.56 ± 0.14 ^e^	1.61 ± 0.17 ^e^	1.93 ± 0.26 ^d^
	GST	88.23 ± 5.66 ^ab^	62.19 ± 4.42 ^c^	92.10 ± 5.07 ^a^	91.77 ± 4.20 ^a^	93.24 ± 6.17 ^a^	83.19 ± 3.53 ^b^	85.04 ± 4.09 ^ab^	86.01 ± 6.38 ^ab^
	GSH	62.08 ± 4.04 ^b^	40.23 ± 2.78 ^e^	79.13 ± 6.11 ^a^	81.25 ± 5.23 ^a^	82.19 ± 4.35 ^a^	48.31 ± 3.96 ^d^	54.19 ± 2.84 ^dc^	55.27 ± 3.36 ^c^

Control: fish fed the basal diet without potassium permanganate exposure; PP: fish fed the basal diet and exposed to potassium permanganate; Cur1: fish fed a diet supplemented with 1% curcumin; Cur2: fish fed a diet supplemented with 2% curcumin; Cur4: fish fed a diet supplemented with 4% curcumin; Cur1+PP: fish fed a diet supplemented with 1% curcumin and subsequently exposed to potassium permanganate; Cur2+PP: fish fed a diet supplemented with 2% curcumin and subsequently exposed to potassium permanganate; Cur4+PP: fish fed a diet supplemented with 4% curcumin and subsequently exposed to potassium permanganate. MDA: Malondialdehyde (nmol/mg protein); SOD: Superoxide dismutase (U/mg protein); CAT: Catalase (k/mg protein); GPx: Glutathione peroxidase (U/mg protein); GST: Glutathione S-transferase (U/mg protein); GSH: Reduced glutathione (µmol/mg protein). Values are presented as mean ± SE (*n* = 30). Different lowercase letters within the same row indicate significant differences among groups (*p* < 0.05, Tukey’s multiple comparison test).

**Table 4:** Effects of dietary curcumin supplementation and potassium permanganate exposure on lipid peroxidation and antioxidant defense system in the kidney.

		Groups							
		Control	PP	Cur1	Cur2	Cur4	Cur1+PP	Cur2+PP	Cur4+PP
Parameters	MDA	1.24 ± 0.12 ^d^	3.81 ± 0.37 ^a^	0.99 ± 0.13 ^e^	0.96 ± 0.09 ^e^	0.95 ± 0.11 ^e^	1.89 ± 0.14 ^b^	1.78 ± 0.17 ^b^	1.51 ± 0.22 ^c^
	SOD	2.85 ± 0.29 ^c^	1.56 ± 0.15 ^e^	3.48 ± 0.41 ^b^	3.99 ± 0.22 ^a^	4.03 ± 0.52 ^a^	2.10 ± 0.19 ^d^	2.21 ± 0.13 ^d^	2.76 ± 0.34 ^c^
	CAT	3.16 ± 0.41 ^c^	1.98 ± 0.32 ^e^	3.89 ± 0.45 ^b^	4.42 ± 0.28 ^a^	4.51 ± 0.37 ^a^	2.68 ± 0.26 ^d^	3.05 ± 0.12 ^c^	3.09 ± 0.30 ^c^
	GPx	2.45 ± 0.18 ^c^	1.17 ± 0.21 ^e^	2.96 ± 0.26 ^b^	3.05 ± 0.33 ^b^	3.68 ± 0.29 ^a^	1.95 ± 0.20 ^d^	2.38 ± 0.39 ^c^	2.42 ± 0.23 ^c^
	GST	101.39 ± 9.74 ^a^	70.36 ± 6.47 ^b^	105.49 ± 7.26 ^a^	108.22 ± 8.14 ^a^	106.39 ± 10.23 ^a^	98.44 ± 6.13 ^a^	96.17 ± 5.76 ^a^	99.91 ± 9.00 ^a^
	GSH	77.24 ± 6.49 ^c^	49.11 ± 5.10 ^f^	91.04 ± 8.32 ^b^	94.26 ± 6.66 ^b^	110.29 ± 7.24 ^a^	60.88 ± 5.29 ^d^	66.27 ± 4.87 ^de^	68.03 ± 6.02 ^e^

Control: fish fed the basal diet without potassium permanganate exposure; PP: fish fed the basal diet and exposed to potassium permanganate; Cur1: fish fed a diet supplemented with 1% curcumin; Cur2: fish fed a diet supplemented with 2% curcumin; Cur4: fish fed a diet supplemented with 4% curcumin; Cur1+PP: fish fed a diet supplemented with 1% curcumin and subsequently exposed to potassium permanganate; Cur2+PP: fish fed a diet supplemented with 2% curcumin and subsequently exposed to potassium permanganate; Cur4+PP: fish fed a diet supplemented with 4% curcumin and subsequently exposed to potassium permanganate. MDA: Malondialdehyde (nmol/mg protein); SOD: Superoxide dismutase (U/mg protein); CAT: Catalase (k/mg protein); GPx: Glutathione peroxidase (U/mg protein); GST: Glutathione S-transferase (U/mg protein); GSH: Reduced glutathione (µmol/mg protein). Values are presented as mean ± SE (*n* = 30). Different lowercase letters within the same row indicate significant differences among groups (*p* < 0.05, Tukey’s multiple comparison test).

**Table 5:** Effects of dietary curcumin supplementation and potassium permanganate exposure on lipid peroxidation and antioxidant defense system in the spleen.

		Groups							
		Control	PP	Cur1	Cur2	Cur4	Cur1+PP	Cur2+PP	Cur4+PP
Parameters	MDA	1.45 ± 0.14 ^d^	2.66 ± 0.23 ^a^	1.28 ± 0.12 ^e^	1.27 ± 0.11 ^e^	1.10 ± 0.16 ^f^	2.19 ± 0.20 ^b^	1.80 ± 0.17 ^c^	1.74 ± 0.32 ^c^
	SOD	2.40 ± 0.22 ^c^	1.62 ± 0.13 ^e^	2.72 ± 0.19 ^b^	2.96 ± 0.15 ^a^	2.99 ± 0.18 ^a^	1.94 ± 0.16 ^d^	2.34 ± 0.14 ^c^	2.35 ± 0.10 ^c^
	CAT	2.55 ± 0.31 ^b^	1.91 ± 0.28 ^d^	2.88 ± 0.33 ^a^	2.94 ± 0.19 ^a^	2.92 ± 0.21 ^a^	2.22 ± 0.14 ^c^	2.25 ± 0.12 ^c^	2.23 ± 0.17 ^c^
	GPx	2.14 ± 0.11 ^c^	1.73 ± 0.20 ^d^	2.47 ± 0.19 ^b^	2.86 ± 0.35 ^a^	2.95 ± 0.39 ^a^	2.05 ± 0.22 ^c^	2.07 ± 0.15 ^c^	2.12 ± 0.28 ^c^
	GST	75.61 ± 4.72 ^ab^	56.29 ± 5.02 ^c^	78.47 ± 4.20 ^ab^	80.16 ± 3.33 ^a^	81.04 ± 4.20 ^a^	70.31 ± 4.18 ^b^	72.19 ± 4.39 ^b^	73.46 ± 3.89 ^b^
	GSH	57.26 ± 3.18 ^c^	41.11 ± 2.74 ^e^	69.13 ± 4.09 ^b^	70.68 ± 4.51 ^b^	79.03 ± 5.17 ^a^	49.24 ± 3.56 ^d^	50.09 ± 4.18 ^d^	51.62 ± 3.72 ^d^

Control: fish fed the basal diet without potassium permanganate exposure; PP: fish fed the basal diet and exposed to potassium permanganate; Cur1: fish fed a diet supplemented with 1% curcumin; Cur2: fish fed a diet supplemented with 2% curcumin; Cur4: fish fed a diet supplemented with 4% curcumin; Cur1+PP: fish fed a diet supplemented with 1% curcumin and subsequently exposed to potassium permanganate; Cur2+PP: fish fed a diet supplemented with 2% curcumin and subsequently exposed to potassium permanganate; Cur4+PP: fish fed a diet supplemented with 4% curcumin and subsequently exposed to potassium permanganate. MDA: Malondialdehyde (nmol/mg protein); SOD: Superoxide dismutase (U/mg protein); CAT: Catalase (k/mg protein); GPx: Glutathione peroxidase (U/mg protein); GST: Glutathione S-transferase (U/mg protein); GSH: Reduced glutathione (µmol/mg protein). Values are presented as mean ± SE (*n* = 30). Different lowercase letters within the same row indicate significant differences among groups (*p* < 0.05, Tukey’s multiple comparison test).

**Table 6:** Effects of dietary curcumin supplementation and potassium permanganate exposure on lipid peroxidation and antioxidant defense system in the gill.

		Groups							
		Control	PP	Cur1	Cur2	Cur4	Cur1+PP	Cur2+PP	Cur4+PP
Parameters	MDA	1.13 ± 0.10 ^f^	3.79 ± 0.62 ^a^	1.41 ± 0.28 ^e^	1.45 ± 0.33 ^e^	1.46 ± 0.24 ^e^	2.23 ± 0.42 ^b^	1.92 ± 0.30 ^c^	1.67 ± 0.21 ^d^
	SOD	1.26 ± 0.14 ^bc^	0.83 ± 0.17 ^d^	1.35 ± 0.09 ^b^	1.36 ± 0.13 ^b^	1.60 ± 0.38 ^a^	1.19 ± 0.26 ^c^	1.23 ± 0.18 ^bc^	1.24 ± 0.10 ^bc^
	CAT	1.63 ± 0.18 ^c^	1.01 ± 0.08 ^f^	1.85 ± 0.23 ^b^	2.10 ± 0.34 ^a^	2.14 ± 0.32 ^a^	1.32 ± 0.19 ^d^	1.33 ± 0.11 ^d^	1.47 ± 0.36 ^e^
	GPx	1.26 ± 0.12 ^c^	0.61 ± 0.09 ^f^	1.55 ± 0.30 ^b^	1.58 ± 0.27 ^b^	1.89 ± 0.41 ^a^	0.89 ± 0.13 ^d^	0.91 ± 0.08 ^d^	1.10 ± 0.11 ^e^
	GST	68.15 ± 3.06 ^a^	52.13 ± 3.47 ^b^	70.75 ± 4.07 ^a^	71.09 ± 3.76 ^a^	72.18 ± 2.93 ^a^	66.53 ± 2.74 ^a^	67.16 ± 3.29 ^a^	66.83 ± 3.77 ^a^
	GSH	62.39 ± 4.20 ^a^	39.21 ± 3.24 ^c^	65.03 ± 3.96 ^a^	64.98 ± 2.78 ^a^	66.27 ± 4.11 ^a^	51.20 ± 3.60 ^b^	60.63 ± 4.29 ^a^	61.02 ± 3.03 ^a^

Control: fish fed the basal diet without potassium permanganate exposure; PP: fish fed the basal diet and exposed to potassium permanganate; Cur1: fish fed a diet supplemented with 1% curcumin; Cur2: fish fed a diet supplemented with 2% curcumin; Cur4: fish fed a diet supplemented with 4% curcumin; Cur1+PP: fish fed a diet supplemented with 1% curcumin and subsequently exposed to potassium permanganate; Cur2+PP: fish fed a diet supplemented with 2% curcumin and subsequently exposed to potassium permanganate; Cur4+PP: fish fed a diet supplemented with 4% curcumin and subsequently exposed to potassium permanganate. MDA: Malondialdehyde (nmol/mg protein); SOD: Superoxide dismutase (U/mg protein); CAT: Catalase (k/mg protein); GPx: Glutathione peroxidase (U/mg protein); GST: Glutathione S-transferase (U/mg protein); GSH: Reduced glutathione (µmol/mg protein). Values are presented as mean ± SE (*n* = 30). Different lowercase letters within the same row indicate significant differences among groups (*p* < 0.05, Tukey’s multiple comparison test).

**Table 7:** Molecular docking scores, interaction types and estimated inhibition constants of Curcumin on SOD, CAT, GPx and GST.

Macromolecule/Pdb ID	Autodock Results				Vina Best Docking Score
	Interacting Residues	Interaction Types	Estimated Inhibition Constant, Ki	Best Docking Score	
SOD/3LSU	C: ALA183 D: HIS30 D: TYR34	Hydrogen Bond Pi-Pi stacking Hydrogen Bond	4.67 µM	−7.27	−7.2
CAT/1DGB	TYR215 TRP303	Hydrogen Bond Hydrogen Bond	1.57 µM	−7.92	−8.8
GPx/2F8A	ASP144 ARG179 ARG180	Hydrogen Bond Pi-cation Hydrogen Bond	151.05 µM	−5.21	−5.7
GST/1F2E	A: ARG35 A: LYS51 A: PRO53 A:GLU65 A: ASN66 A: LYS107	Hydrogen Bond Hydrogen Bond Hydrogen Bond Hydrogen Bond Hydrogen Bond Hydrogen Bond	1.13 µM	−8.11	−7.9

µM: micromolar, Docking Score: kcal/mol.

## Data Availability

The original contributions presented in this study are included in the article. Further inquiries can be directed to the corresponding author.
